# The Role of Sirtuin-1 (SIRT1) in the Physiology and Pathophysiology of the Human Placenta

**DOI:** 10.3390/ijms242216210

**Published:** 2023-11-11

**Authors:** Mateusz Wątroba, Grzegorz Szewczyk, Dariusz Szukiewicz

**Affiliations:** Department of Biophysics, Physiology & Pathophysiology, Medical University of Warsaw, Chałubinskiego 5, 02-004 Warsaw, Poland; mateusz.watroba@wum.edu.pl (M.W.); grzegorz.szewczyk@wum.edu.pl (G.S.)

**Keywords:** sirtuin-1, SIRT1, sirtuins, human placenta, placental physiology, placental pathophysiology, pre-eclampsia, peroxisome proliferator-activated receptor γ, PPARγ, SIRT1/PPARγ signaling

## Abstract

Sirtuins, especially SIRT1, play a significant role in regulating inflammatory response, autophagy, and cell response to oxidative stress. Since their discovery, sirtuins have been regarded as anti-ageing and longevity-promoting enzymes. Sirtuin-regulated processes seem to participate in the most prevalent placental pathologies, such as pre-eclampsia. Furthermore, more and more research studies indicate that SIRT1 may prevent pre-eclampsia development or at least alleviate its manifestations. Having considered this, we reviewed recent studies on the role of sirtuins, especially SIRT1, in processes determining normal or abnormal development and functioning of the placenta.

## 1. Introduction

The placenta is a unique organ occurring during intra-uterine life that plays a significant role in regulating fetal growth and development [1,2]. It consists of trophectoderm-derived epithelial cells, the outer layer of the blastocyst, and extraembryonic mesodermal cells that derive from inner cellular mass—a group of cells that give rise to the embryo proper [3]. In the course of placentation, this combination of cells gives rise to a complex organ that anchors the fetus in the uterine cavity and provides delivery of oxygen, nutrients, and hormones required for fetal growth, as well as the excretion of carbon dioxide and other end products of metabolism. While extra embryonal mesodermal cells give rise to the mesenchymal parts of the placenta, including the fetal circulatory system, trophectoderm-derived epithelial cells differentiate to form two main layers of the trophoblast: villous trophoblast (called the labyrinthine layer in mice) and extravillous trophoblast (called the junctional zone in mice). While the villous trophoblast takes part in gas and nutrient exchange, the extravillous trophoblast anchors the placenta in the uterine wall and remodels maternal spiral arteries to provide sufficient perfusion of the fetoplacental unit [4]. Impaired placental development or function may have significant consequences both for the mother and for the fetus, resulting in complications such as pregnancy-induced hypertension/pre-eclampsia, intra-uterine growth retardation, gestational diabetes, and macrosomia, or may even accelerate the termination of a pathological pregnancy through miscarriage, stillbirth, or preterm birth [5]. Furthermore, some research studies suggest that gestational complications, especially those resulting in intra-uterine growth retardation, can have long-term effects even in postnatal life, contributing to metabolic programming, which can increase the risk of obesity, diabetes, and cardiovascular disease later in life [6]. This is why a deeper understanding of placental development is needed, especially in reference to some signaling pathways that can affect fetal growth.

Sirtuins, a highly conserved group of epigenetic proteins, play an essential role in the comprehensive regulation of metabolic processes at the cellular level. A detailed understanding of their physiological and altered expression in the human placenta may provide valuable information about the physiology of the placenta and the mechanisms of development of placental pathologies. Much of the latest placental research (the results of which we cite and discuss in this review) concerns sirtuin-1 (silent information regulator 2 homolog 1 or SIRT1), a nicotinamide adenine dinucleotide (NAD)-dependent histone deacetylase [1,7,8]. There are seven sirtuins in mammals (SIRT1–SIRT7). All of them deacetylate specific target proteins using NAD+ as a co-substrate, and take part in regulating such processes as oxidative stress response, energy metabolism, inflammatory response, etc. [9]. Several research studies have revealed that sirtuins play a significant role in trophoblast development and differentiation. This does not only apply to SIRT1. For example, SIRT2 is generally expressed in placental syncytiotrophoblast, and its expression is reduced in PE patients. SIRT2 may induce trophoblast cell necrosis while inhibiting trophoblast cell proliferation [10,11]. SIRT3 is reported to affect trophoblast cell migration, invasion, and tube formation, as well as to take part in the pathogenesis of PE [12]. SIRT4 can also induce trophoblast cell senescence [13,14,15]. These results may additionally confirm the hypothesis that SIRT1 deficiency can take part in the pathogenesis of PE by regulating trophoblast cell invasion, migration, and proliferation.

## 2. SIRT1 in the Regulation of Trophoblast Function

### 2.1. Effects towards Placental Development and Differentiation

SIRT1 is crucial for proper trophoblast differentiation and placental development [1,5,7,16,17]. Pre-eclampsia is a hypertensive disorder of pregnancy. It occurs in about 5-8% of all pregnancies. Its symptoms usually appear in the second trimester and comprise arterial hypertension, proteinuria, and edemas. Despite many research studies, detailed aspects of PE pathophysiology have not been completely elucidated, although it is known that the disease is associated with the impaired invasion of extravillous trophoblast into the uterine wall, which results in the impaired remodeling of maternal spiral arteries. This in turn leads to hypoxia, resulting in trophoblast immaturity and compromised angiogenesis within placental villi. Hypoxia, in turn, promotes ROS production, and the related oxidative stress may promote secondary inflammation [18]. This is worth emphasizing, because SIRT1 may support cellular oxidative stress response by activating Nrf2 transcription factors, as well as inhibiting inflammation by deacetylating NF-κB [19]. In placentas and serum samples collected from patients with pre-eclampsia (PE), SIRT1 activity is reduced and can be observed mainly in cell nuclei within cytotrophoblasts and syncytiotrophoblasts [20,21]. SIRT1 probably takes part in trophoblast maintenance and differentiation through modulating small mothers against decapentaplegic proteins 2/3 (SMAD2/3), signal transducer and activator of transcription (STAT), and peroxisome proliferator-activated receptor γ (PPARγ)-dependent signaling pathways [22,23,24,25]. Placentas collected from SIRT1 KO pregnant mice are small and show visible abnormalities within the labyrinthine layer and the junctional zone [20]. In addition, trophoblast stem cells (TSC) collected from SIRT1 knockout (KO) mice show impaired differentiation. In such cells, PPARγ expression is reduced, along with the expression of proteins such as SMAD2, SMAD3, and signal transducer and activator of transcription 3 (STAT3). STAT3 protein is correlated with the differentiation of trophoblast giant cells, while its molecules can be deacetylated by SIRT1, which represses their activity [23,26,27]. Moreover, a possible role of PPARγ in placentation and trophoblast differentiation is emphasized [16,28]. PPARγ activity may be regulated by SIRT1, both through deacetylation and through recruitment of cofactors such as nuclear receptor co-repressor-1 (NCoR1), a silencing mediator of retinoid and thyroid hormone receptors (SMRT), and positive regulatory domain containing 16 (Prdm16) [29,30]. Research studies made so far suggest that the labyrinthine layer of trophoblast is poorly developed in SIRT1+/- mice. At the same time, trophoblast invasive capability is also reduced (even more reduced in SIRT1 KO mice [31]), which may indicate that SIRT1 plays a vital role in placental development and differentiation.

### 2.2. Effects of SIRT1 on Autophagy within Trophoblast

Autophagy is a cell homeostasis-promoting process directing damaged proteins and organelles to lysosomal degradation [32,33,34,35,36]. Autophagy protects the placenta from pathogens and stress. In PE patients, autophagy in trophoblast is impaired, and the accumulation of abnormal proteins within the placenta is increased [37]. Several research studies have shown that SIRT1 prevents H_2_O_2_-induced oxidative stress and apoptosis by promoting autophagy within trophoblast [38]. From a mechanistic standpoint, some studies on autophagy-mediating proteins have shown that SIRT1 may regulate autophagy through NAD^+^-dependent deacetylation of some of them, e.g., transcription factor EB (TFEB), LC3-II (a membrane-bound (lapidated) form of microtubule-associated protein 1 light chain 3 (LC3)), protein that contains a Bcl-2 homology-3 domain (Beclin-1), ubiquitin-binding scaffold protein (p62, also known as sequestosome 1, SQSTM1), and autophagy related-proteins 5, 7, and 8 (ATG5, ATG7, and ATG8, respectively) [39,40]. The formation of lysosomes taking part in autophagy is strictly regulated by TFEB, which can be deacetylated by SIRT1, subsequently activating the expression of several downstream autophagy-related genes, such as lysosomal associated membrane proteins 1 and 2 (*LAMP1*, *LAMP2*) and cathepsin D (*CTSD*) [41,42]. Furthermore, concentrations of protein markers typical for the initial stage of autophagy activation, such as LC-II, Beclin-1, and SQSTM1 [43,44,45], have also been significantly altered in the course of PE and can be regulated by SIRT1 [46,47,48]. These results, taken together, suggest that SIRT1 may regulate autophagy within the trophoblast through deacetylation of its specific target proteins taking part in the process of autophagy.

### 2.3. Effects on Cell Senescence Phenotype Occurrence within the Placenta

Premature senescence of the placenta is a trait typical for PE. It manifests with increased occurrence of cell senescence phenotype (CSP), senescence-associated secretory phenotype (SASP), and enhanced expression of cell senescence markers such as the protein encoded by the TP53 tumor suppressor gene (p53) and cyclin-dependent kinase inhibitor p21 (p21). Loss of SIRT1 activity is also a specific marker of cell senescence, and SIRT1 deficiency results in premature senescence within placentas during their formation [49,50,51,52]. Interestingly, Xiong et al. found that reduced SIRT1 activity promotes p53 acetylation and p21 expression, as well as impairs trophoblast cell migration and invasion in case of advanced maternal age, which suggests that SIRT1 deficiency can take part in the pathogenesis of PE by inducing CSP within the placenta.

## 3. SIRT1 and PPARγ

Peroxisome proliferator-activated receptor γ (PPARγ) belongs to the family of nuclear hormonal ligand-activated receptors. It can also act as a transcription factor, widely known because of its crucial role in glucose and lipid metabolism, as well as in adipocyte differentiation. After dimerization with retinoid X-receptor (RXR), PPARγ binds specific DNA sequences defined as PPARγ-reactive elements (PPRE) and subsequently induces genes involved in fatty acid assimilation and accumulation, which results in lipid accumulation and adipogenesis.

PPARγ is necessary for forming white and brown adipose tissue, with white adipose tissue being the site of energy storage and hormone secretion. In contrast, brown adipose tissue is the site of energy expenditure and thermogenesis. PPARγ may be activated by thiazolidinediones (TZDs) (synthetic activators that are sometimes used to treat type 2 diabetes mellitus) [53,54].

Sirtuin 1 (SIRT1) is a member of NAD+-dependent protein deacetylases, at the same time functioning as a sensor of cell nutritional status. Its orthologue has been initially discovered in budding yeast Saccharomyces cerevisiae as a longevity-promoting enzyme. SIRT1 has been first identified as a histone deacetylase, promoting chromatin compaction and silencing transcription of some genes in case of undernutrition [55]. However, recent studies have identified numerous non-histone substrates of SIRT1, including p53, forkhead O class box transcription factors (FoxOs), and PPARγ. By deacetylating PPARγ, SIRT1 renders its inactivation, thus inhibiting adipogenesis and promoting fat mobilization at the same time.

On the other hand, the inhibition of SIRT1 expression with small interfering RNA (siRNA) promotes adipogenesis and inhibits lipolysis [56]. In addition to its effects on metabolism, SIRT1 regulates many other signaling pathways, including those involved in cell proliferation, apoptosis, autophagy, and inflammatory response [57,58]. SIRT1 can be activated by the naturally occurring compound resveratrol—identified as an anti-inflammatory and anti-oxidative agent—and by small molecule synthetic activators [59].

Although SIRT1 inhibitory action towards PPARγ has been studied quite well, the interaction between these two proteins is not so simple. It has been found that PPARγ deacetylation by SIRT1 results in the recruitment of a positive regulatory (PR) domain zinc finger protein 1 (PRDM1) coactivator, which selectively activates PPARγ to stimulate the conversion of white adipose tissue to brown adipose tissue [60]. Furthermore, PPARγ can also be an upstream inhibitor of SIRT1, both by inhibiting its deacetylase activity and its expression at the level of transcription [61]. Finally, both thiazolidinedione PPARγ activators and SIRT1 activators, such as resveratrol, may exert collateral effects. TZDs induce a transient overexpression of SIRT1 [62], while resveratrol binds some nuclear receptors from the PPAR family, including PPARγ [63]. Thus, evaluating cross-talk between signaling pathways dependent on these two proteins requires a thorough analysis of experiment results, especially if activators of both proteins are used.

### 3.1. Role of SIRT1- and PPARγ-Dependent Signaling Pathways in Placental Pathology

Impaired trophoblast differentiation and placental development are correlated with many complications of pregnancy, including miscarriage, pre-eclampsia, intra-uterine growth retardation, and gestational diabetes [64,65,66]. These complications are related to a suboptimal microenvironment at the maternal side of the placenta, showing signs of hypoxia, oxidative stress, inflammation, and/or hyperglycemia. This is why it should be stated how those alterations in the placental microenvironment may affect SIRT1 and PPARγ-dependent signaling in the placenta.

#### 3.1.1. Effects of Hypoxia on PPARγ Activity

Oxygen tension is an essential parameter within the placenta, both in the course of normal development and in specific placental pathologies [67]. During hypoxia, i.e., when oxygen tension is too low, many signaling pathways are activated, subsequently affecting tissue homeostasis. Relatively best known among them is a signaling pathway activated by hypoxia-inducible factor (HIF), which is a complex of two component proteins: hypoxia-inducible factor subunit alpha (HIFα) domain that is stabilized with oxygen and hypoxia-inducible factor subunit beta (HIFβ) domain that is expressed constitutively [68]. An HIF-dependent signaling pathway is necessary for both placental formation and development, especially for trophoblast differentiation to invasive cell lines (trophoblast giant cells in mice, corresponding to human extravillous trophoblast) [69,70,71]. It is known that hypoxia affects PPARγ activity through the HIF complex. Thus, hypoxia inhibits adipocyte differentiation through its effect on HIF-dependent PPARγ2 (an isoform of PPARγ typical for adipose tissue) [72]. In mouse trophoblast stem cells, hypoxia inhibits PPARγ activity, but this effect is independent of HIF activity. [73]. In addition, forced PPARγ expression during hypoxia may, in part, rescue trophoblast cell differentiation into the labyrinthine layer in mice [73].

The results presented above correlate with hypoxia-associated placental pathology that occurs in the course of pre-eclampsia at the maternal site. Abnormal differentiation of syncytiotrophoblast, which is an analogue of the labyrinthine layer of trophoblast in mice, is a typical feature of this condition, regarded as secondary to the reduced blood supply to the maternal part of the placenta because of abnormal remodeling of spiral arteries by invasive cells of extravillous trophoblast [5]. Placentas collected from PE patients show a reduced expression of PPARγ and decreased activity of glial cells missing-1 (GCM1)—a primary regulator of syncytiotrophoblast formation and probably a target protein for PPARγ. GCM1 can, in turn, activate protein referred to as syncytin-1 [74,75,76], and trophoblast abnormalities similar to those occurring during PE have been recapitulated in vitro by repressing the level of GCM1 [77]. Therefore, it is presumed that reduced PPARγ activity due to hypoxia may inhibit GCM1 and syncytin-1 expression, thus negatively affecting syncytiotrophoblast differentiation.

Another finding typical for PE is an elevated level of anti-angiogenic molecules—soluble vascular endothelial growth factor (VEGF) receptors, also known as soluble fms-like tyrosine kinase 1 (sFlt-1)—in maternal blood [78]. Although the etiology and origin of increased sFlt-1 release from placentas in PE patients is still debated, several studies have shown a correlation between hypoxia and increased sFlt-1 expression in human trophoblast [79,80,81,82]. Some studies reveal a correlation between sFlt-1 levels in syncytiotrophoblast and the severity of PE manifestations [83,84]. PPARγ activity has been negatively correlated with increased s-Flt-1 levels in the rat model of PE. Pregnant female rats show PE symptoms, such as arterial hypertension, proteinuria, and fetal growth retardation, when treated with a PPARγ antagonist. These symptoms are associated with an increased sFlt-1 concentration in the plasma [85]. Interestingly, one study on mice showed a correlation between a reduced level of GCM1 and a raised level of sFlt-1 in the plasma [86]. The combined results of these studies suggest that the PPARγ-GCM1 axis can regulate sFlt-1 expression. When evaluating the levels of sFlt-1 mRNA and sFlt-1 release from differentiated mouse trophoblast stem cells (TSC) after their treatment with PPARγ activator (rosiglitazone), reduced levels of both sFlt-1 mRNA and sFlt-1 release can be found. Rosiglitazone does not affect sFlt-1 levels in wild-type TSC (WT TSC) exposed to hypoxia and in PPARγ KO TSC exposed to normoxia or hypoxia, which suggests that the observed effect is PPARγ-dependent.

Finally, PE is characterized by increased apoptosis in trophoblast cells [87], which is interesting because PPARγ is one of the molecules involved in apoptosis. When trophoblast cells are cultured under hypoxic conditions, their differentiation to form syncytiotrophoblast is impaired, and severe hypoxia leads to apoptosis [88,89]. In similar conditions, treating these cells with the PPARγ activator (rosiglitazone) promotes their normal differentiation and alleviates apoptotic damage [88]. These results combined may be a premise regarding PPARγ as a potential target protein in the treatment of placental pathologies, such as PE.

#### 3.1.2. Effects of Hypoxia on SIRT1 Activity

Compared with correlations between hypoxia and PPARγ activity, the correlation between hypoxia and SIRT1 activity seems more complex. In several research studies, SIRT1 has been identified as an upstream regulator of HIFα domains. SIRT1 may deacetylate hypoxia-inducible factor 1 subunit alpha (HIF-1α), which results in the blocked recruitment of p300 domains and thus abrogates the expression of HIF-1α effector genes [90]. In addition, SIRT1 selectively stimulates the activity of hypoxia-inducible factor 2 subunit alpha (HIF-2α), thus promoting hypoxia-related signaling dependent on this alternative HIFα domain [91]. Moreover, SIRT1 gene expression can be impaired by hypoxia in an HIF-dependent manner, which suggests a feedback loop between these two proteins [92]. SIRT1 expression within trophoblast under hypoxic conditions has not been evaluated in detail. However, one study has shown SIRT1 induction during hypoxia in human trophoblast cells, which results in the upregulation of N-myc downstream-regulated gene 1 (*NDRG1*) and reduced expression of p53, thus promoting cell survival [93]. Nevertheless, more detailed studies are necessary for pinpointing precise and detailed correlations between hypoxia, HIF activity, and SIRT1 activity, both in the trophoblast and the placenta.

Similarly to PPARγ, SIRT1 activity is reduced within syncytiotrophoblast isolated from PE patients’ placentas [94]. It is hypothesized that this result is related to increased CSP occurrence among trophoblast cells during PE, especially when taking considered SIRT1 longevity-promoting functions [94]. In several studies, SIRT1 activity has also been negatively correlated with PE manifestations. In one of them, the SIRT1 activator resveratrol has been shown to inhibit sFlt-1 release induced by treating human placentas with tumor necrosis factor-alpha (TNF-α) or by exposing them to hypoxia. Resveratrol also reduces sFlt-1 release from placental explants collected from PE patients, although only by 25–30% [95]. In a recent study, the exposure of primary human trophoblast cells to resveratrol has been found to inhibit both sFlt-1 secretion and sFlt-1 mRNA transcription [96]. Also, in differentiated mouse WT TSC and SIRT KO TSC, treatment with resveratrol inhibits both sFlt-1 secretion and sFlt-1 mRNA transcription in a SIRT1-dependent manner. Since PPARγ activity also falls with SIRT1 KO [20], there is probably more than one target protein through which SIRT1 regulates basic levels of sFlt-1. Finally, resveratrol has also been reported to alleviate arterial hypertension and proteinuria in rat models of PE [97]. These data suggest that, although SIRT1 is required to maintain low expression of sFlt-1, it inhibits sFlt-1 release from cells. Thus, SIRT1 activation may be regarded as a therapeutic option in PE patients, along with PPARγ activation.

#### 3.1.3. Effects of SIRT1 and PPARγ Action towards Placentas Exposed to Oxidative Stress

Increased oxidative stress levels have also been found in placentas collected from PE patients [98]. This means there is an excess of reactive oxygen species (ROS), which may be secondary to hypoxia, ischemia/reperfusion, or reduced levels of anti-oxidants. Since oxidative stress as a possible effect of hypoxia has already been discussed, this paragraph will focus on other possible causes of oxidative stress. In the rat model of maternal malnutrition, a diet deficient in folic acid and cobalamin applied in pregnant females results in an increased level of oxidative stress markers in the plasma and in reduced levels of PPARγ mRNA in the placenta. However, it has no consequences for fetuses or mothers, unless the placental mass is affected [99]. As to SIRT1, oxidative stress induced by the treatment of human placental explants with hypoxanthine/xanthine oxidase reduces both SIRT1 mRNA and protein expression, as well as inhibits the expression of glucose transporter 1 (GLUT1), a glucose transporter responsible for glucose uptake [21]. These phenotypic alterations can be abrogated by resveratrol [21].

**Figure 1:**
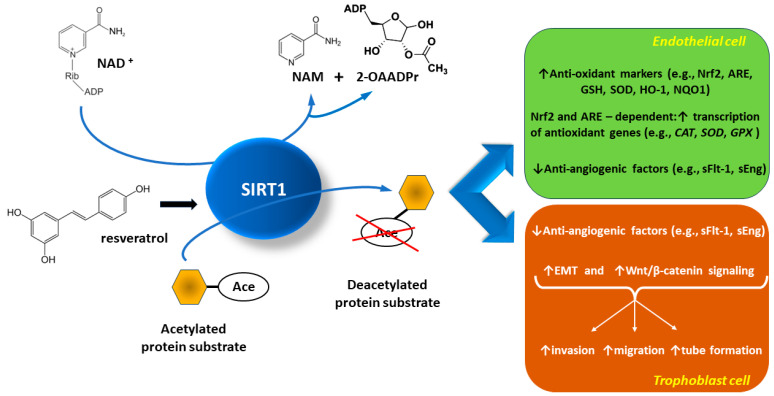
Mechanisms of the beneficial effect of the natural sirtuin-1 activator resveratrol at the endothelium level and trophoblast level under oxidative stress (e.g., accompanying pre-eclampsia) [7,101,102,103,104,105].

Similarly, treatment with resveratrol reduces oxidative stress in the placenta, as well as apoptosis occurrence in rat models of PE induced with L-NG-nitroarginine methyl ester (L-NAME) [97]. RNA profiling in mouse TSC has identified glutathione peroxidase isoform-encoding genes (*GPX1* and *GPX2*) in ten genes most repressed in SIRT1 KO TSC. The GPX protein family accounts for cell protection from oxidative stress by catalyzing the reduction of organic hydroperoxides and hydrogen peroxide with glutathione (GSH) [100]. The reduced expression of GPX1 and GPX3 in SIRT1 KO TSC in comparison with WT TSC has been confirmed with quantitative polymerase chain reaction (qPCR) and correlated with increased apoptosis occurrence in SIRT1 KO TSC. It should be a subject of further evaluation whether SIRT1 KO TSC is indeed more susceptible to oxidative stress because of reduced glutathione peroxidase (GPX) expression. A sum of resveratrol actions as an SIRT1 activator is depicted in Figure 1.

Sirtuin-1 (SIRT1) acts primarily by removing acetyl groups from lysine residues within substrate proteins in the presence of nicotinamide adenine diphosphate (NAD+). The NAD+ dependence determines that the levels of NAD+ and SIRT1 activity (deacetylation) are tightly coupled. The acetyl group is transferred to the 2′-OH position of ADP-ribose, ultimately yielding nicotinamide (NAM) and 2′-O-acetyl-ADP-ribose (2-OAADPr). This oxidative stress-induced epigenetic mechanism reveals/increases the expression of genes that counteract pre-eclampsia by reducing hypertension, oxidative stress, inflammation, and apoptosis in the placenta.

The anti-hypertensive effect of resveratrol through the activation of SIRT1 at the placental level in pre-eclampsia is mainly based on the inhibition of anti-angiogenic factors, soluble fms-like tyrosine kinase-1 (sFlt-1), and soluble endoglin (sEng), which are known to cause endothelial and trophoblast dysfunction. In addition, SIRT1 reduces the expression of pro-inflammatory molecules and increases the expression of anti-oxidant molecules in endothelial cells. Such endothelial anti-oxidant markers in the pre-eclamptic placenta are nuclear factor erythroid 2-related factor 2 (Nrf2), anti-oxidant response element (ARE), glutathione (GSH), superoxide dismutase (SOD), heme oxygenase-1 (HO-1), and NADPH-quinone oxidoreductase-1 (NQO1). The Nrf2-ARE pathway is an intrinsic mechanism of defence against oxidative stress. Its activation in endothelial cells triggers the transcription of anti-oxidant genes, encoding, among others, catalase (*CAT*), *SOD,* and glutathione peroxidase (*GPX*).

Increased SIRT1 activity in the pre-eclampsia placenta may promote trophoblast cell invasion, migration, and tube formation. This is achieved by activating epithelial–mesenchymal transition (EMT) and the Wnt/β-catenin pathway. Wnt/β-catenin signaling, a highly conserved pathway through evolution, regulates vital cellular functions, including proliferation, differentiation, migration, genetic stability, apoptosis, and stem cell renewal.

#### 3.1.4. Effects of SIRT1 and PPARγ towards Placentas Affected by Inflammatory Response

Inflammatory response within the placenta can occur within the frames of physiology or pathology. An example of a physiologic inflammatory response is the one observed within the placenta and fetal membranes during standard delivery [106]. Such pro-inflammatory conditions at delivery have been correlated with unchanged PPARγ expression accompanied by reduced SIRT1 expression both in fetal membranes and the placenta [21,107]. Pro-inflammatory cytokines regulate SIRT1 expression in the human placenta and its level has been reported to fall after the exposal on interleukin-1 beta (IL-1β) and TNF-α [21]. Quite interestingly, visfatin/nicotinamide mononucleotide adenyltransferase (Nampt), an adipokine and SIRT1 activator, positively correlates with SIRT1 activity, and its level rises in obese women’s placentas just before delivery, which may suggest a possible mechanism preventing SIRT1 activity falling during late pregnancy. It can sometimes be responsible for post-term delivery, commonly observed in obese pregnant women [108].

Pathologic inflammatory response within the placenta correlates with PE and maternal obesity [109,110]. In the case of micro-element deficiency, pronounced inflammation within the placenta has been correlated with reduced expression of PPARγ mRNA in pregnant female rats [111]. On a mouse model of lipopolysaccharide (LPS)-induced intra-uterine fetal death (IUFD), the preliminary treatment of pregnant mice with PPARγ activator rosiglitazone reduced IUFD occurrence from 64% to 16% [112]. This effect is related to the enhanced nuclear location of PPARγ within placental trophoblast cells, as well as to the reduced expression of placental pro-inflammatory mediators, such as interleukin-6 (IL-6) and TNF-α, and abrogating LPS-induced nuclear translocation of PPARγ within the labyrinthine layer of the trophoblast [112]. Finally, on a rat model of LPS-induced PE, a transplant of human mesenchymal stem cells (MSC) resulted in reduced activity of pro-inflammatory mediators, such as IL-6 and TNF-α, as well as increased placental PPARγ activity; this was accompanied by a milder course of arterial hypertension and greater fetal mass in comparison with rats treated with LPS alone [113].

Much less is known about the correlation between obesity-related inflammation within the placenta and SIRT1/PPARγ expression. This type of inflammation is characterised by T lymphocyte and macrophage infiltration within chorionic villi. This type of inflammation occurs twice as often within the placentas of female fetuses, although the reason why is unknown [114]. While macrophage infiltration within adipose tissue has been correlated with reduced SIRT1 expression [115], no alterations in SIRT1 expression have been reported in placentas collected from obese mothers. However, decreased placental SIRT1 expression accompanied by increased placental PPARγ expression can be observed in a mouse model on a high fat diet during pregnancy [20]. It has been correlated with increased activity of placental lipoprotein lipase (LPL), as well as with increased adipose tissue content in fetuses, which suggests that maternal overnutrition affects fetal development through altering the activity of SIRT1 and PPARγ [116]. The dependence of these phenotypic traits on pro-inflammatory mediators has yet to be elucidated. Reduced SIRT1 activity has been reported in mouse WT TSC treated with IL-6 [1], but the way it is correlated with other markers of trophoblast cell differentiation has not been evaluated.

#### 3.1.5. Correlations between Hyperglycemia and Placental SIRT1/PPARγ Activity

Although no studies have been conducted on alterations of placental SIRT1 activity in the case of maternal diabetes mellitus, similar studies referring to placental PPARγ activity have shown exciting results. PPARγ activity has been increased in human primary trophoblast cells exposed to hyperglycemia [117] and in the placentas of pregnant female mice with streptozotocin-induced diabetes mellitus [118]. On the other hand, many other studies have observed reduced placental PPARγ expression in the case of gestational diabetes mellitus [119,120,121,122], and one of those studies has revealed reduced expression of this protein both in syncytiotrophoblast and in extravillous trophoblast [121]. It should be emphasized that pregnancies complicated with gestational diabetes mellitus are characterized by pronounced inflammation within the placenta [123,124], which is important since some pro-inflammatory cytokines affect PPARγ expression [125]. Further studies are necessary to precisely evaluate the correlation between gestational diabetes mellitus and PPARγ activity within the trophoblast and placenta, considering both the management of maternal hyperglycemia and related effects towards fetal growth.

## 4. SIRT1-Dependent Prevention of Pre-Eclampsia

### 4.1. SIRT1 Protective Actions towards Vascular Endothelial Cells

**Figure 2:**
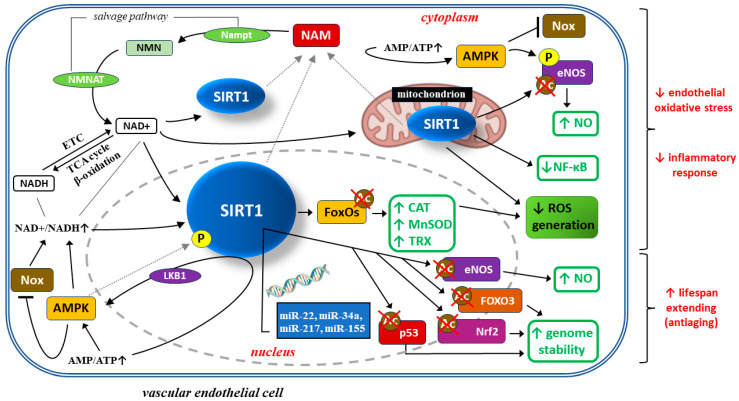
Beneficial effects of SIRT1 on vascular endothelial cells in pre-eclampsia: limiting oxidative stress, inflammatory response, and cellular ageing.

Endothelial cell dysfunction is one of the typical traits of pre-eclampsia, resulting from several factors, including oxidative stress, inflammatory response, autophagy, etc. SIRT1 counteracts oxidative stress and exerts some anti-inflammatory and anti-aging effects. Several research studies have shown that SIRT1 activity is reduced in serum samples collected from PE patients and in human umbilical vein endothelial cells (HUVEC) incubated with such serum [126]. SIRT1 may protect HUVEC from necrosis in PE patients, thus blocking PE development [127]. From the mechanistic point of view, SIRT1 protects endothelial cells from oxidative stress, inflammatory response, and cell senescence phenotype through numerous mechanisms, as depicted in Figure 2.

SIRT1 (silent information regulator 2 homolog 1) is a crucial cellular survival protein, especially in oxidative stress environments. SIRT1 activity depends on the oxidized form of nicotinamide adenine dinucleotide (NAD+), which is generated from its precursor—nicotinamide mononucleotide (NMN)—by enzyme nicotinamide-(mono)nucleotide adenylyltransferase (NMNAT). Three NMNAT isoforms have been discovered, and they show distinct subcellular localizations—NMNAT1 (nucleus), NMNAT2 (cytosol), and NMNAT3 (mitochondria)—which suggests a localization component to NAD+ synthesis in response to metabolic signals [128,129]. Similarly, although the nucleus is the main leading site of SIRT1 synthesis, its activity is also observed in the cytoplasm and mitochondria [128,130]. The level of NAD+ is determined by NAD+ synthesis from the salvage pathway or NAD+/reduced form (NADH) ratio. Mitochondrial redox metabolism within the electron transport chain (ETC) is crucial for SIRT1 levels because NAD+/NADH and AMP/ATP metabolism results from the tricarboxylic acid (TCA) cycle and β-oxidation or oxidative phosphorylation, respectively [131]. NAD+ is required in the SIRT1-mediated deacetylase reaction. This reaction also generates nicotinamide (NAM), which enters the salvage pathway. Nicotinamide mononucleotide adenyltransferase (Nampt) catalyses the conversion from NAM to NMN and is a thrate-limiting enzyme in this pathway. NMN is thereby converted to NAD+ by NMNAT.

The NAD+/NADH ratio and AMP/ATP ratio increase during caloric restriction and are well-known inducers of SIRT1.

SIRT1 attenuates oxidative stress and inflammation to regulate vascular endothelial functions through several important signal mediators, such as AMP-activated protein kinase (AMPK), nicotinamide adenine dinucleotide phosphate (NADPH) oxidase (Nox), endothelial nitric oxide synthase (eNOS), and forkhead transcription factors of the O class (FOXOs) [7,132]. SIRT1 can stimulate AMPK via the modulation of upstream AMPK kinases such as liver kinase B1(LKB1), suppressing the production of reactive oxygen species (ROS) and inflammatory response in human umbilical vein endothelial cells (HUVECs). At the same time, AMPK influences SIRT1 deacetylation activity by increasing cellular NAD+ levels or directly phosphorylating (P) SIRT1. An increased AMP/ATP ratio induces endothelial AMPK, which in turn suppresses Nox expression and Nox-induced ROS production [133]. AMPK-dependent phosphorylation and SIRT1-dependent deacetylation of eNOS leads to an increase in local nitric oxide (NO) concentration. Moreover, SIRT1 deacetylates FoxO proteins and thus stimulates FoxO-dependent anti-oxidative enzymes, such as catalase (CAT), manganese superoxide dismutase (MnSOD), and thioredoxin (TRX), eliminating ROS from endothelial cells and thus preventing endothelial dysfunction [132,134,135]. SIRT1 protects endothelial cells from senescence by regulating signaling pathways dependent on tumor supressor protein p53 (p53), eNOS, transcription factor nuclear factor erythroid 2-related factor 2 (Nrf2), and FOXO3. Expression of these proteins can be regulated at the translation level by several micro-RNA molecules, such as mi-R217, mi-R34a, mi-R155, and mi-R22 [136,137,138,139,140,141,142]. Optimization of NO concentration and genome stability extend the average lifespan of endothelial cells.

#### 4.1.1. SIRT1 and the Protection of Endothelial Cells against Oxidative Stress and Inflammatory Response

Oxidative stress and inflammatory response are mutually related pathophysiologic processes taking part in the pathogenesis of PE. Oxidative stress consists of raised ROS concentrations, which results in an inflammatory response and, in the case of endothelial cells, their damage and dysfunction [7]. Mitochondrial function is impaired during PE, which results in increased ROS generation, mainly in the form of superoxide anions, causing oxidative stress and systemic inflammation [143,144,145,146]. SIRT1 inhibition abrogates endogenous anti-oxidative systems’ activity in in vitro PE models. Furthermore, SIRT1 is necessary for counteracting oxidative stress and inflammation in diabetic angiopathy [147,148,149], while the same two phenomena (i.e., oxidative stress and inflammation) play a crucial role in the pathogenesis of PE. SIRT1 inhibition in hyperglycemic conditions results in endothelial cell dysfunction, while SIRT1 activation alleviates endothelial aging induced by oxidative stress in diabetic mice [150,151]. Quite interestingly, SIRT1 alleviates oxidative stress and inflammatory response by regulating endothelial cell functions via several signaling pathways dependent on adenosine monophosphate(AMP)-activated protein kinase (AMPK), nicotinamide adenine dinucleotide phosphate (NADPH) oxidases (Nox), endothelial nitric oxide synthase (eNOS), and FoxOs [152]. There is a complex network of interactions between AMPK and SIRT1. SIRT1 may activate AMPK by modulation of liver kinase B1 (LKB1) (an upstream regulatory enzyme, modulating AMPK activity [152,153]), which inhibits ROS production and inflammatory response in HUVEC. AMPK also affects SIRT1 deacetylase activity by regulating intra-cellular NAD+ concentration or directly phosphorylating SIRT1 molecules. In addition, increased activity of NADPH oxidase (Nox) can also increase intra-cellular NAD+ concentration, which stimulates SIRT1 activity in endothelial cells [132].

Moreover, SIRT1 deacetylates FoxO proteins and thus stimulates FoxO-dependent antioxidative enzymes, such as catalase (CAT), manganese superoxide dismutase (MnSOD), and thioredoxin (TRX), eliminating ROS from endothelial cells and thus preventing endothelial dysfunction [132,133,134,135]. SIRT1 has been reported to stimulate c-Myc expression by promoting forkhead box protein O1 (FoxO1) degradation, which prevents hyperglycemia-induced endothelial cell dysfunction and angiogenesis [154]. eNOs, as a nitric oxide synthase (NOS) family protein, is expressed in vascular smooth muscle cells. It plays a crucial role in the pathogenesis of PE by catalyzing nitric oxide (NO) biosynthesis while inhibiting ROS production [155]. SIRT1 may directly deacetylate eNOs, or stimulate eNOs activity indirectly by affecting FoxO proteins and AMPK-dependent signaling pathways [156], which can participate in PE pathogenesis. This evidence suggests that SIRT1 may protect endothelial cells from oxidative stress and inflammatory response through interacting with other enzymes, which can take part in the pathogenesis of PE.

#### 4.1.2. SIRT1 May Protect Endothelial Cells through Autophagy Regulation

**Figure 3:**
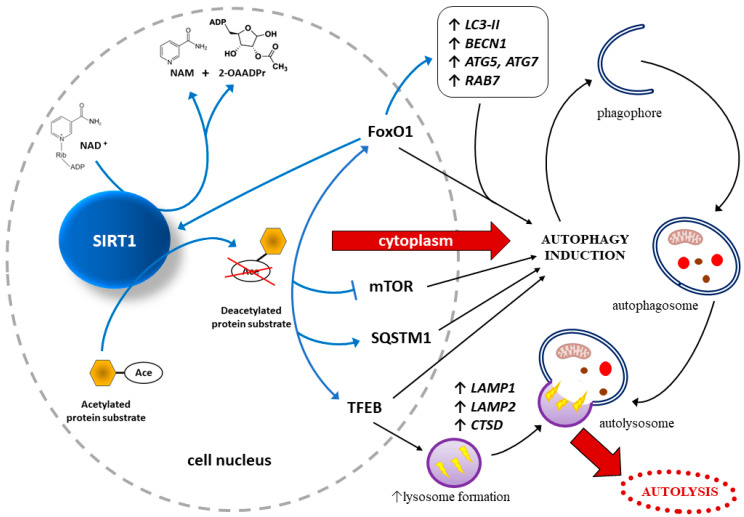
Mechanisms of action of SIRT1 towards maintaining the homeostasis of both endothelial cells and trophoblast by promoting autophagy.

In endothelial cells, autophagy is regulated mainly by SIRT1-dependent and FoxO-dependent signaling pathways, which may take part in the pathogenesis of PE [157]. Research studies have found that SIRT1 activates FoxO_1_, thus protecting endothelial cells through autophagy regulation [158]. To be more precise, SIRT can deacetylate and thus activate FoxO_1_, while activated FoxO_1_ may stimulate SIRT1 expression [159]. FoxO_1_ is strictly related to autophagy since it modulates the expression of such proteins taking part in autophagy as a small GTPase Rab7, LC3, ATG-5, and Beclin-1 [160]. These results suggest that SIRT1 exerts a protective effect on endothelial cells, analogously to the trophoblast (see Section 2.2. Effects of SIRT1 on Autophagy within Trophoblast), also by regulating autophagy via multiple signaling pathways, as shown in Figure 3.

Sirtuin-1 (SIRT1) acts as an energy and redox sensor because it is activated by nicotinamide adenine dinucleotide (NAD^+^), an essential substrate in energy and oxidation reactions. As a result of NAD^+^-dependent deacetylation of the respective protein substrates, acetyl groups are transferred to the 2′-OH position of ADP-ribose, ultimately yielding nicotinamide (NAM) and 2′-O-acetyl-ADP-ribose (2-OAADPr) [128,129]. There is an increase in the level of gene expression and, consequently, proteins responsible for the induction and promotion of autophagy, such as forkhead box protein O1 (FoxO1), sequestosome 1 (SQSTM1, also known as ubiquitin-binding scaffold protein p62), and transcription factor EB (TFEB) (a key regulator of the autophagy/lysosomal-to-nucleus signaling pathway) [46]. Moreover, the formation of lysosomes is strictly regulated by TFEB via activation of several downstream autophagy-related genes, such as lysosomal associated membrane protein 1 and 2 (*LAMP1*, *LAMP2*) and cathepsin D (*CTSD*) [41,42].

Sirt1-deacetylated FOXO1 stimulates the expression of *RAB7*, encoding a small GTPase that is a crucial factor in the maturation of autophagosomes and endosomes [161]. Other autophagy-related genes activated directly by SIRT1 or via FoxO1 are those that encode the membrane-bound lipidated form of LC3 (*LC3-II*), a protein containing a Bcl-2 homology-3 domain (*BECN1*), and autophagy related-proteins 5 and 7 (*ATG5*, *ATG7*) [39,40]. In addition, FoxO1 directly activates SIRT1, thus creating an autofeedback loop regulating SIRT1 expression [49]. Autophagy is also induced by SIRT1 inhibiting the mammalian target of the rapamycin (mTOR)-related signaling pathway [162,163]. Cell autolysis as a result of autophagy is preceded by the formation of phagophore, autophagosome, and, after fusion of the autophagosome and a lysosome, autolysosome [32,33,34,35,36].

#### 4.1.3. SIRT1 and Possible Protection of Endothelial Cells against Senescence

CSP occurrence in vascular endothelial cells is a direct cause of the most dangerous complications of cardiovascular diseases and, thus, the most frequent cause of death [164,165]. Quite interestingly, in PE patients, CSP has been observed in endothelial progenitor cells, which is related to endothelial dysfunction [166,167]. SIRT1 protects endothelial cells from CSP by regulating some signaling pathways dependent on p53, eNOs, Nrf2, forkhead box protein O3 (FoxO3), and p21/p16. Expression of these proteins can be regulated at the translation level by several micro-RNA molecules, such as mi-R217, mi-R34a, mi-R155, and mi-R22 [136,137,138,139,140,141,142]. Although additional research studies may be required to pinpoint the SIRT1 role in endothelial cell senescence phenotype regulation precisely, hitherto performed studies suggest that SIRT1 protects endothelial cells from oxidative stress, inflammatory response, CSP, and autophagy through deacetylation of its specific target proteins, which may take part in the pathogenesis of PE.

### 4.2. Anti-Inflammatory Action of SIRT1 within the Placenta in the Context of Pre-Eclampsia

SIRT1 is essential in alleviating inflammatory response and oxidative stress in several physiologic and pathologic conditions [168]. Reduced placental SIRT1 expression means that anti-inflammatory and anti-oxidative protection has been compromised. In addition, SIRT1 inhibits high mobility group box 1 (HMGB1) release from cells to the extra-cellular space through deacetylation of this non-histone protein molecule. Although, in most cases, HMGB1 binds to DNA and promotes transcription of its specific target genes, it is not sensu stricto nuclear protein, and its molecules can be translocated to other organelles or even actively released from cells, which is usually induced by exposal to specific factors (e.g., hypoxia). HMGB1 molecules can also be passively released from cells following cell necrosis [169]. Extra-cellular HMGB1 activates nuclear factor kappa-light-chain-enhancer of activated B cells (NF-κB) through interactions with receptors for advanced glycation end products (RAGE) and toll-like receptor 4 (TLR4), as well as promotes secretion of pro-inflammatory cytokines, such as TNF-α, IL-1, IL-6, and interleukin-8 (IL-8) [169]. Therefore, HMGB1 release to the extra-cellular environment activates innate and adaptive immunity. Moreover, recent research studies suggest that HMGB1 released from hypoxic trophoblast may increase endothelial permeability through signaling pathways dependent on TLR4 and caveolin-1 [170]. Increased endothelium permeability is the main cause of proteinuria and generalized edemas in the course of PE [170]. HMGB1 concentration in HUVEC-containing medium rises after HUVEC treatment with IL-6 or with serum collected from PE patients despite reduced HMGB1 concentration in these cells, which suggests that HMGB1 is released from the cells in such conditions. Furthermore, experiments comprising SIRT1 inhibition or activation have shown that SIRT1 may block HMGB1 release from cells on a mouse model of PE, which suggests that SIRT1 can abrogate pro-inflammatory actions of HMGB1 in the course of PE [127]. In addition, SIRT1 inhibits 70-kDa heat shock protein (HSP70) release from HUVEC after their exposal to IL-6 or serum collected from PE patients. In cells unexposed to stress, HSP70 undergoes a constitutive expression and plays many significant physiologic roles in almost every organelle, including cytoplasm, endoplasmic reticulum, mitochondria, and cell nucleus [171]. Several kinds of stress induce HSP70 expression and, initially, it has been thought that it helps the cells counteract the stress [171]. However, more recent studies have revealed that HSP70 released to the extra-cellular environment may bind to many specific signaling receptors, such as lectin-like oxidized low-density lipoprotein-1 (LOX-1), toll-like receptor 2 (TLR2), TLR4, 50-kDa integral membrane protein of the tumor necrosis factor receptor (TNF-R) family (CD40), scavenger receptor expressed by endothelial cell-1 (SREC-1), and link domain-containing scavenger receptor-1 (FEEL-1), which implicates the ambiguous effects of HSP70 in some conditions [171]. It has been confirmed that HSP70 activates human monocytes, inhibiting the release of some pro-inflammatory cytokines, such as TNF-α, IL-1β, IL-6, and interleukin-10 (IL-10). However, another study has revealed that, in patients suffering from early-onset PE, HSP70 concentration in the plasma is positively correlated with the concentration of TNF-α, soluble type 1 receptor for TNF-α, IL-1β, interleukin-12 (IL-12), glutamicoxaloacetic transaminase (GOT), glutamic pyruvic transaminase (GPT), lactate dehydrogenase (LDH), and uric acid, which in turn suggests that a raised level of HSP70 is related to an increased negative effect towards maternal and fetal well-being [171,172]. Molvarec et al. [173] have delivered more evidence, suggesting that HSP70 may contribute to systemic inflammatory responses in PE patients. They have found the serum HSP70 level to be positively correlated with increased levels of such proteins as interleukin-12 subunit beta p40 (IL-12p40), monocyte chemoattractant protein-1 (MCP-1), intercellular adhesion molecule 1 (ICAM-1), and vascular cell adhesion molecule 1 (VCAM-1). They have also shown that raised levels of HSP70 and sFlt-1/placenta growth factor (PlGF) are independent risk factors of PE development. Although the pathogenic functions of HSP70 in PE remain controversial, HUVEC releases HSP70 to the extra-cellular environment in response to stimulation with IL-6 or serum collected from PE patients, which suggests that they perceive such stimulation as a kind of stress. The forced expression of SIRT1 inhibits HSP70 release from cells, which indicates that it may inhibit HUVEC response to stress in the course of PE. One recent research study has shown that SIRT1 affects HSP70 expression in cells. Inducible HSP70 is upregulated in the spinal cord of mice chronically overexpressing SIRT1 in the central nervous system [174]. Studies on SIRT1 mechanisms of action indicate that it deacetylates heat shock transcription factor 1 (HSF-1), a transcription factor that is an essential regulator of heat shock protein (HSP) expression, which results in an enhanced expression of inducible HSP70 [174]. Few studies, however, deal with SIRT1’s role in modulating HSP70 release from cells. Those studies that have explored this issue may deliver the first piece of evidence that SIRT1 can inhibit HSP70 release from HUVEC cells, thus counteracting the effects of exposal to IL-6 or serum collected from PE patients. However, further studies are necessary to pinpoint the mechanisms through which SIRT1 modulates HSP70 release from cells. During PE, excessive inflammatory response and oxidative stress result in endothelial cell damage and death. Necrotic cells may release HMGB1, further enhancing inflammatory response, resulting in a vicious circle. SIRT1 protects HUVEC from necrosis resulting from exposal to IL-6 or serum collected from PE patients. This protective effect of SIRT1 is probably related to its anti-inflammatory and anti-oxidative actions and to its anti-apoptotic functions. Many studies have confirmed that SIRT1 can deacetylate p53, thus abrogating its pro-apoptotic actions [175]. Furthermore, SIRT1 has been found to be downregulated in the placentas of PE model mice, while HMGB1 and HSP70 serum concentrations are markedly elevated in such mice. SIRT1 inhibits HMGB1 and HSP70 release from HUVEC exposed to IL-6 or serum collected from PE patients and protects the cells from necrosis. All these findings indicate that SIRT1 can play a protective role in PE, alleviating its manifestations.

### 4.3. SIRT1 Alleviates PE Course on Animal Models of PE

SIRT1 activity is reduced in PE patients’ placentas and sera and in placentas and sera collected from mice used in an animal model of PE [176]. It has been found that SIRT1 inhibition in SIRT1^+/−^ mice induces typical manifestations of PE, such as arterial hypertension, proteinuria, intra-uterine growth retardation, renal damage, as well as labyrinthine layer atrophy. Moreover, all of these manifestations can be alleviated by the treatment with the experimental drug SRT2104, a potent SIRT1 inducer [31]. It has also been reported that SIRT1 KO mice placentas and fetuses show abnormalities both within the labyrinthine layer and in the junctional zone. In addition, SIRT1 KO mice develop numerous abnormalities (from increased prenatal mortality to fetal growth impairment), resulting in more significant postnatal mortality [177,178,179,180]. Furthermore, placentas collected from SIRT1 KO mice show increased occurrence of cell senescence phenotype, as well as other morphologic abnormalities [181], which is strictly correlated with PE development.

### 4.4. SIRT1 Induction Alleviates PE Manifestations

In reduced uterine perfusion pressure (RUPP) rats, constituting an animal model of PE, supplementation with recombined SIRT1 protein alleviates PE manifestations, such as arterial hypertension, impaired placental angiogenesis, inflammatory response, and unfavorable pregnancy outcome [182]. Similar effects may be achieved through treatment with the SIRT1 inducer SRT2104 [31]. However, more animal studies and clinical trials are needed to precisely determine SIRT1’s role in PE.

## 5. Conclusions

Recently, there have been more and more publications on the activity of sirtuins in placental tissue in normal and complicated pregnancies. This applies in particular to SIRT1 in placental vascular endothelial cells and trophoblast cells [8,183,184,185,186]. Directly involved in many key intra-cellular reactions, SIRT1 builds up the connection between epigenetics and metabolism at the placental level [187]. As an NAD+-dependent deacetylase, SIRT1 regulates many aspects of chromatin biology, such as transcription, recombination, and genome stability, by modifying histones, transcription factors, and epigenetic enzymes, and, in connection with the above, it directly affects placental homeostasis by modifying a diverse set of metabolic enzymes, both in the cytosol and in the mitochondria [1,188]. The beneficial effects of SIRT1 in the human placenta known so far relate primarily to modulating the activity of factors responsible for the course of the inflammatory response, oxidative stress, autophagy, and cell senescence [7,38,160,189,190,191,192].

Therefore, there are well-documented reasons to treat the activation or inhibition of SIRT1 as a potential therapeutic target, especially in hypertensive disorders complicating pregnancy, in which the main pathomechanism is based on endothelial dysfunction [5,7,8,193]. This is even more important as the incidence of PE increases, possibly as a result of increased prevalence of predisposing disorders, such as chronic hypertension, diabetes, and obesity [194,195].

## Data Availability

Not applicable. This review is based on already published data listed in the references.

## Abbreviations

2-OAADPr	2′-O-acetyl-adenosine diphosphate(ADP)-ribose.
AMPK	adenosine monophosphate(AMP)-activated protein kinase.
ARE	antioxidant response element.
ATG5: ATG7, ATG8	autophagy related-proteins 5, 7, 8.
Beclin-1	the mammalian ortholog of yeast Atg6/Vps30, an essential autophagy protein that contains a Bcl-2 homology-3 domain.
*BECN1*	Beclin-1 gene.
*CAT*: CAT	catalase gene, catalase (anti-oxidant enzyme), respectively.
CD40	50-kDa integral membrane protein of the tumor necrosis factor receptor (TNF-R) family.
CSP	cell senescence phenotype.
CTSD	cathepsin D.
EMT	epithelial–mesenchymal transition.
eNOS	endothelial nitric oxide synthase.
ETC	electron transport chain.
FEEL-1	link domain-containing scavenger receptor-1.
FoxOs	forkhead O class box transcription factors.
FoxO1: FoxO3	forkhead box protein O1 and O3, respectively.
GCM1	glial cells missing-1 (transcription factor).
GLUT1	glucose transporter 1.
GOT	glutamicoxaloacetic transaminase.
GPT	glutamic pyruvic transaminase.
GPX	glutathione peroxidase.
GPX1: GPX2, GPX3	glutathione peroxidase isoforms 1, 2, and 3, respectively.
GSH	glutathione.
H_2_O_2_	hydrogen peroxide.
HIF	hypoxia-inducible factor (HIF).
HIF-1α	hypoxia-inducible factor 1 subunit alpha.
HIF-2α	hypoxia-inducible factor 2 subunit alpha.
HIFα: HIFβ	domains that make up the (hypoxia-inducible factor) HIF molecule domain.
HMGB1	high mobility group box 1 (non-histone nuclear protein).
HO-1	heme oxygenase-1.
HSF1	heat shock transcription factor 1.
HSP70	70-kDa heat shock proteins.
HSPs	heat shock proteins.
HUVEC	human umbilical vein endothelial cells.
ICAM-1	inter-cellular adhesion molecule 1, also known as CD54 (cluster of differentiation 54).
IL-1: IL-1β, IL-6, IL-8, IL-10, IL-12	interleukins: 1, 1 beta, 6, 8, 10, and 12.
IUFD	intra-uterine fetal death.
IL-12p40	interleukin-12 subunit beta (p40).
KO	knockout.
L-NAME	L-NG-nitroarginine methyl ester.
LAMP1: LAMP2	lysosomal associated membrane protein 1,2.
LC3	microtubule-associated protein 1 light chain 3 (MAP1LC3), a human homologue of yeast Atg8, an essential component of autophagy.
LC3-II	membrane-bound: lipidated form of LC3.
LDH	lactate dehydrogenase.
LKB1	liver kinase B1.
LOX-1	lectin-like oxidized low-density lipoprotein-1.
LPL	placental lipoprotein lipase.
LPS	lipopolysaccharide.
MCP-1	monocyte chemoattractant protein-1.
mi-R217: mi-R34a, mi-R155, mi-R22	micro-RNA molecules.
MnSOD	manganese superoxide dismutase (antioxidant enzyme).
mTOR	mammalian target of rapamycin (an ubiquitous serine-threonine protein kinase).
NAD	nicotinamide adenine dinucleotide.
NAD^+^	nicotinamide adenine dinucleotide (oxidized form).
NADH	nicotinamide adenine dinucleotide (reduced form, H for hydrogen).
NAM	nicotinamide.
NCoR1	nuclear receptor co-repressor-1.
*NDRG1*	N-myc downstream-regulated gene 1 (formerly known as Drg1, Cap43, Rit42, *RTP*, and *PROXY-1*).
Nampt	nicotinamide mononucleotide adenyltransferase.
NF-κB	nuclear factor kappa-light-chain-enhancer of activated B cells.
NMN	nicotinamide mononucleotide.
NMNAT	nicotinamide-(mono)nucleotide adenylyltransferase.
NMNAT1: NMNAT2, NMNAT3	nicotinamide-(mono)nucleotide adenylyltransferase isoforms: 1,2, and 3.
NO	nitric oxide.
NOS	nitric oxide synthase.
Nox	nicotinamide adenine dinucleotide phosphate (NADPH) oxidases.
NQO1	nicotinamide adenine dinucleotide phosphate (NADPH)-quinone oxidoreductase-1.
Nrf2	nuclear factor erythroid 2-related factor 2.
p62	ubiquitin-binding scaffold protein, also known as sequestosome 1 (SQSTM1).
p53	protein encoded by the *TP53* tumor suppressor gene, marker of cell senescence.
p21	cyclin-dependent kinase inhibitor p21, protein marker of cell senescence.
PE	pre-eclampsia.
PlGF	placenta growth factor.
PPARγ	peroxisome proliferator-activated receptor γ.
PPARγ2	an isoform of PPARγ typical for adipose tissue.
PPRE	PPARγ-reactive elements.
PRDM1	positive regulatory (PR) domain zinc finger protein 1, a coactivator selectively activating PPARγ.
Prdm16	positive regulatory domain containing 16
qPCR	quantitative polymerase chain reaction.
Rab7	a small GTPase: member of the Rab family that controls transport to late endocytic compartments such as late endosomes and lysosomes.
*RAB7*	Rab7 gene.
RAGE	receptor for advanced glycation end-products.
ROS	reactive oxygen species.
RUPP	reduced uterine perfusion pressure.
RXR	retinoid X-receptor.
SASP	senescence-associated secretory phenotype.
sEng	soluble endoglin: the extracellular domain of membrane endoglin.
sFlt-1	soluble fms-like tyrosine kinase 1, also known as soluble vascular endothelial growth factor (VEGF) receptor-1.
siRNA	small interfering RNA.
SIRT1	silent information regulator 2 homolog 1 or sirtuin-1.
SIRT7	sirtuins 1 to 7.
SMAD2: SMAD3	small mothers against decapentaplegic proteins 2 and 3 (transcription factors).
SMRT	silencing mediator of retinoid and thyroid hormone receptors.
SOD	superoxide dismutase.
SQSTM1	sequestosome 1: also known as ubiquitin-binding scaffold protein p62.
SREC-1	scavenger receptor expressed by endothelial cell-1.
SRT2104	experimental drug, a selective small molecule activator of SIRT1.
STAT	signal transducer and activator of transcription (transcription factor).
STAT3	signal transducer and activator of transcription 3 (transcription factor).
TCA	tricarboxylic acid cycle, also known as the Krebs cycle or the citric acid cycle.
TFEB	transcription factor EB (TFEB), a member of the MiT/TFE family of basic helix-loop-helix leucine zipper transcription factors, a key regulator of the autophagy/lysosomal-to-nucleus signaling pathway.
TLR2: TLR4	toll-like receptor 2 and 4.
TNF-α	tumor necrosis factor alpha.
TRX	thioredoxin (anti-oxidant protein).
TSC	trophoblast stem cells.
TZD	thiazolidinediones: synthetic activators of PPARγ.
WT TSC	wild-type trophoblast stem cells (TSC).
VCAM-1	vascular cell adhesion molecule 1, also known as CD106 (cluster of differentiation 106).
VEGF	vascular endothelial growth factor.
